# Mapping Pulmonary and Systemic Inflammation in Preschool Aged Children With Cystic Fibrosis

**DOI:** 10.3389/fimmu.2021.733217

**Published:** 2021-10-15

**Authors:** Shivanthan Shanthikumar, Sarath C. Ranganathan, Richard Saffery, Melanie R. Neeland

**Affiliations:** ^1^ Infection and Immunity Theme, Murdoch Children’s Research Institute, Parkville, VIC, Australia; ^2^ Department of Paediatrics, The University of Melbourne, Parkville, VIC, Australia; ^3^ Respiratory and Sleep Medicine, Royal Children’s Hospital, Parkville, VIC, Australia

**Keywords:** paediatrics, flow cytometry, lung diseases, cystic fibrosis, immune profiling, BAL, respiratory

## Abstract

The immune landscape of the paediatric respiratory system remains largely uncharacterised and as a result, the mechanisms of globally important childhood respiratory diseases remain poorly understood. In this work, we used high parameter flow cytometry and inflammatory cytokine profiling to map the local [bronchoalveolar lavage (BAL)] and systemic (whole blood) immune response in preschool aged children with cystic fibrosis (CF) and aged-matched healthy controls. We demonstrate that children with CF show pulmonary infiltration of CD66b^+^ granulocytes and increased levels of MIP-1α, MIG, MCP-1, IL-8, and IL-6 in BAL relative to healthy control children. Proportions of systemic neutrophils positively correlated with age in children with CF, whilst systemic CD4 T cells and B cells were inversely associated with age. Inflammatory cells in the BAL from both CF and healthy children expressed higher levels of activation and migration markers relative to their systemic counterparts. This work highlights the utility of multiplex immune profiling and advanced analytical pipelines to understand mechanisms of lung disease in childhood.

## Introduction

A detailed understanding of the tissue-specific immune landscape in health and disease is required to improve the clinical management of many childhood diseases. Aberrant inflammation is a hallmark of several childhood lung diseases, including cystic fibrosis (1), bronchopulmonary dysplasia (2), preschool wheeze (3), asthma (4), primary ciliary dyskinesia (5), and COVID-19 (6). Despite this, little is known regarding the immune cell profiles and mechanisms governing inflammatory processes in the early life respiratory system.

A major limitation in defining immune cell development in the paediatric lung has been the availability of tissue samples collected in early life. Unlike adults, children infrequently undergo surgical procedures for evaluation of lung diseases, and as such research samples are not readily obtained. One clinical test which can be leveraged for research purposes in children is the bronchoalveolar lavage (BAL), which samples immune cells in the lung. Furthermore, the recent advancement of multiple single cell technologies, including single-cell RNA sequencing (sc-RNAseq), single cell DNA methylation analysis (7), and single cell Assay for Transposase-Accessible Chromatin sequencing (8), along with improvements in existing techniques such as flow cytometry and CyTOF, now mean that small volumes of childhood BAL fluid (collected at the time of clinically indicated procedures) can be used to profile the immune cells and inflammatory factors of the lung in highly granular detail. These technologies are complemented by new unsupervised analysis tools, including hierarchical clustering [e.g. Seurat (9), FlowSOM (10)] and dimensionality reduction [e.g. UMAP (11), tSNE (12)] algorithms that reduce reliance on prior knowledge and assumptions, and permit unbiased assessment of high dimensional data. Consequently, unsupervised analyses are increasingly viewed as the gold standard assessment of single cell data

We previously published a flow cytometry-based protocol for immune phenotyping of cryopreserved BAL fluid from children with CF (13). This work revealed that the most common immune cell population in the paediatric lung is the alveolar macrophage, comprising up to 90% of immune cells in BAL. This was followed by granulocytes, making up approximately 5% of immune cells, as well as small proportions of lymphocytes, monocytes, and dendritic cells. This was the first report to provide a detailed assessment of immune cell frequencies from childhood lung samples, however it was limited by its use of cryopreserved samples and a lack of a healthy control reference. A previous study developed a flow cytometry panel for immune cell assessment of nasopharyngeal aspirate and tracheal aspirate samples from children (14). However, this report did not include markers for alveolar macrophages, monocytes, dendritic cells, or B cells, nor an assessment of the proportions of immune cell types identified in these samples.

In the present study, we sought to provide comprehensive experimental and analytical methods for immune cell and cytokine profiling of the early life respiratory system in children, using samples collected from children with cystic fibrosis and aged-matched healthy controls. We performed simultaneous assessment of fresh BAL fluid and whole blood for comparisons between circulating and tissue-resident immune cell profiles, analysed functional markers associated with cell activation and migration, and applied unsupervised clustering and visualisation analysis tools to both lung and blood datasets. This is the first description of simultaneous use of these methods for paediatric respiratory samples and highlight their utility in developing a better understanding of the role of inflammation in lung diseases of childhood.

## Materials and Methods

### Study Participants

All subjects (CF and healthy controls) are enrolled in the AREST CF cohort at the Royal Children’s Hospital, Melbourne, Australia (15). **Supplementary Table 1** describes the demographics of the study participants. All families gave written and informed consent for their involvement in the AREST CF research program (HREC #25054), which includes collection of samples and clinical data. Healthy controls were children with no history of lower airway disease who underwent bronchoscopy to investigate upper airway pathology such as stridor.

### Sample Collection

All subjects underwent clinically indicated bronchoscopy. Bronchoalveolar lavage (BAL) was performed under general anaesthesia. Each BAL aliquot consisted of 1mL/kg (maximum 20mL) of normal saline being inserted *via* the working channel of the bronchoscope and then suctioned for return. BAL samples were placed on ice and processed for flow cytometry within 1 hour of the procedure. Venous blood samples were collected in EDTA tubes from each participant at the time of BAL collection. Blood samples were kept at room temperature and processed for flow cytometry within 1 hour of collection.

### Flow Cytometry of BAL and Whole Blood

BAL samples were centrifuged at 300 x g for 10 mins at 4°C. Cell-free BAL supernatant was then collected and stored at -80°C. The cell pellet was resuspended in 10mL of media [RPMI supplemented with 2% foetal calf serum (FCS)], filtered through a 70uM filter and centrifuged at 300 x g for 10 mins at 4°C. Supernatant was discarded and the cell pellet resuspended in PBS for viability staining using near infra-red viability dye according to manufacturers’ instructions. Following blood collection, 100 µl of EDTA whole blood was aliquoted for flow cytometry analysis and lysed with 1mL of red cell lysis buffer for 10 mins at room temperature. Cells were washed with 1mL PBS and centrifuged at 400 x g for 5 mins. Following another wash, cells were resuspended in PBS for viability staining using near infra-red viability dye according to manufacturers’ instructions. For both BAL and whole blood samples, the viability dye reaction was stopped by the addition of FACS buffer (2% heat-inactivated FCS in 2mM EDTA) and cells were centrifuged at 400 x g for 5 mins. Cells were then resuspended in human FC-block according to manufacturers’ instructions for 5 minutes at room temperature. The antibody cocktail (**Supplementary Table 2**) made up at 2X concentration was added 1:1 with the cells and incubated for 30 minutes on ice. Following staining, cells were washed with 2 mL FACS buffer and centrifuged at 400 x g for 5 minutes. Cells were then resuspended in 2% PFA for a 20 minute fixation on ice, washed, and resuspended in 150µl FACS buffer for acquisition using the BD LSR X-20 Fortessa and BD FACS DIVA V 9.0 software. For all flow cytometry experiments, compensation was performed at the time of sample acquisition using compensation beads. **Supplementary Figure 1** depicts the manual gating strategy for BAL samples. **Supplementary Figure 2** depicts the manual gating strategy for whole blood samples.

### Quantification of Inflammatory Cytokines in BAL and Whole Blood

Cell-free BAL supernatant and plasma samples were thawed and cytokines were assessed using the Human Soluble Protein Flex Set Cytometric Bead Array (BD Biosciences) according to the manufacturer’s instructions. Cytometric bead array data were acquired on an LSR II X-20 Fortessa and analysed using the FCAP Array Software. Both sample types were assessed for the following 10 cytokines: IL-1β, IL-6, IL-8, MCP-1, MIP-1α, MIG, RANTES, TNFα, IFNα, and IL-12p70. Cytokine levels below the detection range, as supplied by the manufacturer, were arbitrarily reported as half the lower limit of detection for each cytokine and included in the analysis. IL-12p70 was undetectable in all BAL samples and was excluded from further analysis for BAL samples.

### Data Analysis

**Figure 1:**
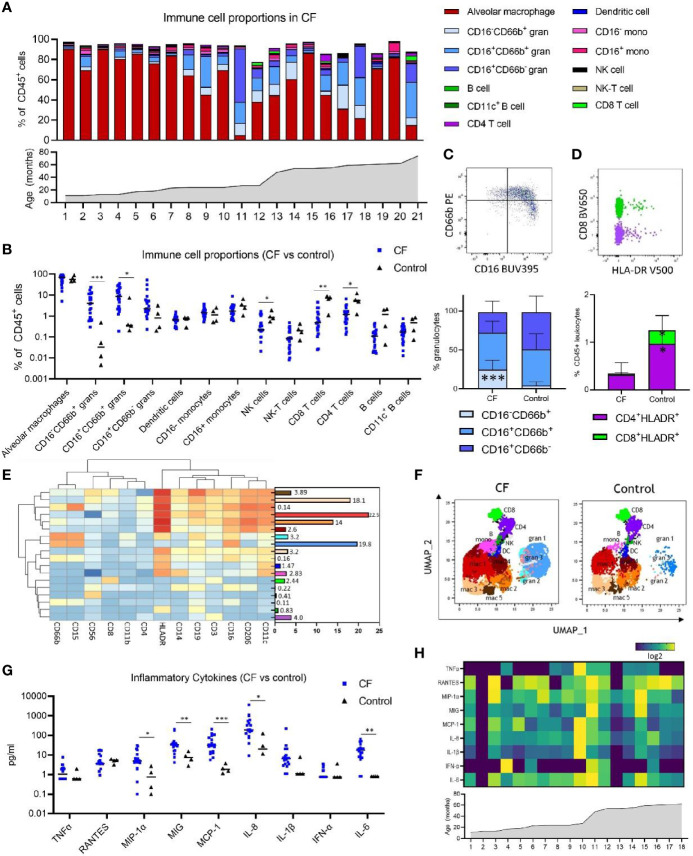
Immune cell and inflammatory cytokine profile of early life bronchoalveolar lavage (BAL) samples from children with cystic fibrosis (CF) and healthy controls. **(A)** Frequencies of BAL immune cell subpopulations expressed as percentage of CD45^+^ leukocytes and in ascending order of age in children with CF (n = 21). **(B)** Frequencies of immune cell subpopulations in BAL of children with CF (n = 21) and healthy controls (n = 4). **(C)** Granulocyte subpopulations in BAL of CF and control children, represented by a flow cytometry plot and the bar graph beneath **(D)** HLA-DR^+^ CD4 and CD8 T cells in BAL of CF and control children, represented by a flow cytometry plot and the bar graph beneath. **(E)** FlowSOM clustering of BAL using 13 lineage markers revealed 18 cell clusters which have been annotated based on expression pattern and projected onto a **(F)** UMAP plot with colours corresponding to the annotated FlowSOM clusters. **(G)** Concentration of inflammatory cytokines in BAL of children with CF (n = 18) and healthy controls (n = 4). **(H)** Heatmap depicting individual BAL cytokine responses in 18 children with CF in ascending order of age. All p-values by Mann-Whitney U test, *p < 0.05, **p < 0.01, ***p < 0.001.

**Figure 2:**
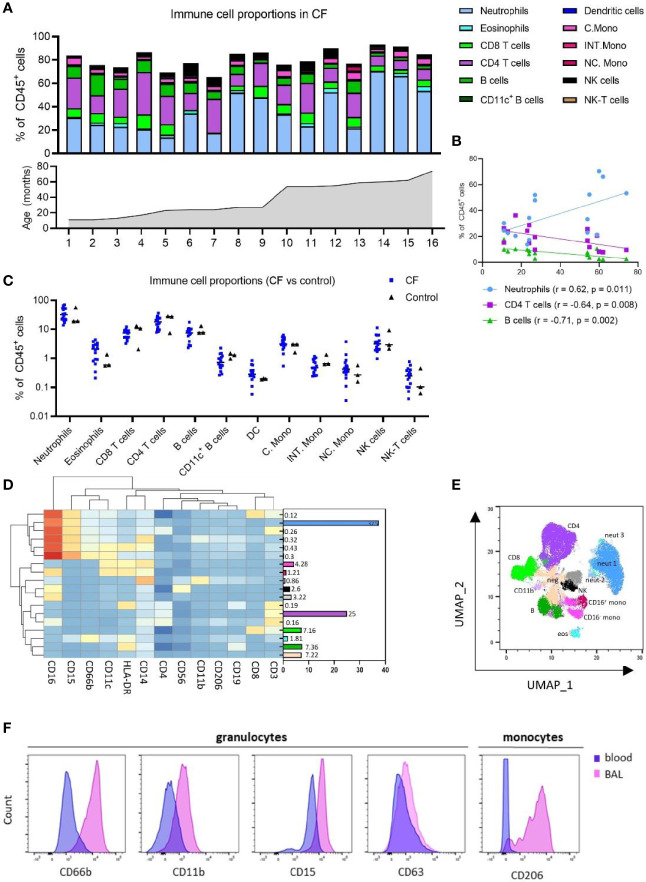
Immune cell and inflammatory cytokine profile of early life blood samples from children with cystic fibrosis (CF) and healthy controls. **(A)** Frequencies of peripheral blood immune cell subpopulations expressed as percentage of CD45^+^ leukocytes and in ascending order of age in children with CF (n = 16). **(B)** Two-sided spearman correlation analysis of circulating neutrophils, CD4 T cells and B cells with age in children with CF. **(C)** Frequencies of immune cell subpopulations in peripheral blood of children with CF (n = 16) and healthy controls (n = 3). **(D)** FlowSOM clustering of blood using 13 lineage markers revealed 18 cell clusters which have been annotated based on expression pattern and projected onto a **(E)** UMAP plot with colours corresponding to the annotated FlowSOM clusters. **(F)** Expression profiles of granulocytes and CD16^+^ monocytes in BAL and blood for activation and migratory markers CD66b, CD11b, CD15, CD63 and CD206.

Flow cytometry results were analysed (manual gating, FlowSOM, UMAP) using FlowJo Version 10.8. software. Manual gating was performed according to the gating strategy depicted in **Supplementary Figures 1, 2**, using both unstained and FMO-controls. FlowSOM and UMAP analyses of BAL and blood data were conducted using a concatenated file for each sample type containing 5000 randomly selected live single CD45^+^ cells per individual. Algorithms were run using default parameters within FlowJo. For FlowSOM, a target of 18 meta-clusters was selected for both blood and BAL samples after extensive preliminary iterations. Manually gated results are presented as proportion of CD45^+^ leukocytes. Flow cytometry and cytokine data were plotted in Prism version 9.1. Individual data points are shown. For **Figures 1C, D**, mean and standard deviation are shown. The heatmap in **Figure 1H** depicts the log2 BAL cytokine value for each cytokine and individual. For statistical comparisons between CF and healthy controls (**Figures 1B, G**, **2C**), Mann-Whitney U tests were performed. For correlations (**Figure 2B**), two-sided spearman’s rank tests were performed. All statistical testing was performed using Prism version 9.1 and p<0.05 was considered significant.

## Results

### Immune Cell Profile of BAL During the First Six Years of Life in Children With CF

BAL samples from 25 children aged between 1-6 years (n=21 with CF, n=4 healthy controls) were used in this study for respiratory analysis (**Supplementary Table 1**). Blood samples from a subset of these children (n=16 with CF, n=3 healthy controls) were used for systemic analysis (**Supplementary Table 1**). A single flow cytometry panel consisting of 16 antibodies was used for both BAL and whole blood (**Supplementary Table 1**). Manual gating analysis using the gating strategy outlined in **Supplementary Figure 1** was used to determine the cellular immune profile of paediatric BAL (**Figure 1A**).

Alveolar macrophages were the predominant immune cell type in BAL of children with CF at a median of 69.4% of CD45^+^ leukocytes. The BAL granulocyte fraction was subtyped based on CD16 and CD66b granulocyte marker expression, with CD16^-^CD66b^+^ granulocytes comprising a median of 4.02% of leukocytes, CD16^+^CD66b^+^ granulocytes comprising 8.45% of leukocytes, and CD16^+^CD66b^-^ granulocytes comprising 2.2% of leukocytes (**Figure 1A** and **Supplementary Figure 3A**). A CD16^-^CD66b^-^ population was not identified (**Supplementary Figure 1**). Monocytes were defined based on CD14 and CD16^+/-^ expression, with approximately equal proportions of CD16^+^ and CD16^-^ monocytes observed in BAL of children with CF (1.71 and 1.42%, respectively) (**Figure 1A** and **Supplementary Figure 3A**). Dendritic cells, NK cells, NK-T cells, CD8 T cells, CD4 T cells, and B cells were also identified in BAL, totalling to a median of 2.78% of leukocytes combined. The median cell proportions for each of these cell types (expressed as percentage of leukocytes) were 0.65%, 0.22%, 0.08%, 0.55%, 1.18% and 0.10%, respectively (**Figure 1A** and **Supplementary Figure 3A**). A subset of CD19^+^CD11c^+^ B cells were also identified, comprising a median of 0.17% of immune cells in BAL (**Supplementary Figure 3A**).

As we had analysed samples from children aged 1-6, we next sought to investigate changes in cell frequency across the first five years of life in CF. **Figure 1A** shows individual cell proportions in ascending order of age, highlighting the interpatient variability of lung immune cells in CF. CD16^+^CD66b^-^ granulocytes were the only pulmonary cell population significantly associated with age (spearman r = 0,67, p=0.0008) (**Supplementary Figure 3B**).

### Pulmonary Granulocyte Infiltration and T Cell Activation in CF

Relative to healthy control children, CF children demonstrate elevated inflammatory cellular and cytokine signatures in the lung (**Figures 1B–G**). BAL samples from children with CF show significantly higher levels of both subsets of CD66b^+^ granulocytes [CD16^-^CD66b^+^ (median 4.02% vs 0.03%, p=0.0003) and CD16^+^CD66b^-^ (median 8.4% vs 0.35%, p=0.03)] (**Figure 1B**). This increase in granulocytes was also reflected in a proportionate decrease in the frequency of lymphocytes in BAL of children with CF relative to healthy controls [NK cells (median 0.22% vs 0.86%, p = 0.024), CD8 T cells (median 0.55% vs 7.19%, p = 0.0041), and CD4 T cells (median 1.18% vs 5.4%, p = 0.024).

Within the granulocyte population itself, CF children show higher frequency of the CD16^-^CD66b^+^ granulocytes relative to healthy control children (median 22.61% vs 2.54%, p = 0.0002) (**Figure 1C**), reflecting redistribution of the granulocyte subsets in addition to an increase in their total frequency.

T cell activation was also assessed by analysing expression of HLA-DR on CD4 and CD8 T cells, revealing that CF children show lower proportions of HLA-DR^+^ CD4 and CD8 T cells relative to healthy control children [HLADR^+^ CD4 (median 0.24% vs 1.04%, p = 0.03), HLADR^+^ CD8 (median 0.01% vs 0.23%, p = 0.014)] (**Figure 1D**).

### Replication of Findings Using Unsupervised Analysis of Flow Cytometry Data

To confirm the findings observed by manual gating and explore the immune profile of our BAL in more detail, we performed unsupervised clustering analysis of BAL data using FlowSOM (10). To visualise these data in two dimensions, the non-linear dimensionality reduction technique UMAP (11) was applied and the cells colour highlighted by their respective FlowSOM cluster (**Figures 1E, F**). Clustering analysis using 13 lineage markers revealed 18 cell clusters (**Figure 1E**). Based on the expression of lineage markers, the clusters were classified into annotated cell types. This revealed clusters corresponding to six alveolar macrophage populations, three granulocyte populations, two monocyte populations, and one cluster corresponding to each of NK cells, CD8 T cells, CD4 T cells, dendritic cells, and B cells. The average frequencies of each cluster across all BAL samples are shown in **Figure 1E**. Clusters corresponding to alveolar macrophage populations were highly positive for CD206, CD11c and HLA-DR, whilst demonstrating an intrinsically auto-fluorescent signature, as previously reported (16). Clusters corresponding to granulocyte populations all expressed CD15 and CD66b, with granulocyte cluster 2 also expressing HLA-DR, and the most abundant granulocyte cluster 1 expressing high levels of CD16. Monocyte clusters expressed characteristic markers such as CD11c, HLA-DR and CD14, however one monocyte population also expressed CD206 and CD16.

To further verify our clustering results, the UMAP analysis depicted in **Figure 1F** shows that FlowSOM clusters corresponding to macrophage populations, granulocyte populations, monocytes, dendritic cells, NK cells, CD4 T cells, CD8 T cells and B cells cluster together as expected within two-dimensional space. Furthermore, a comparison of UMAP plots between CF and control samples show differences in the proportions of granulocyte clusters, reflective of what was observed in the manual gating analysis. Cell proportions obtained from FlowSOM analysis highly correlate with those obtained using manual gating (**Supplementary Figure 3C**). UMAPs for each individual are depicted in **Supplementary Figure 3D**.

### Elevated Inflammatory Cytokines in BAL of Children With CF

We next explored the inflammatory cytokine profile of BAL in CF and control participants. Children with CF show increased levels of MIP-1α, MIG, MCP-1, IL-8, and IL-6 in BAL relative to healthy control children (**Figure 1G**) [MIP-1α (4.9pg/ml vs 0.75pg/ml, p = 0.01), MIG (33.81pg/ml vs 7.41pg/ml, p = 0.007), MCP-1 (31.14pg/ml vs 1.8pg/ml, p=0.0003), IL-8 (191.3pg/ml vs 20.32pg/ml, p=0.01), and IL-6 (17.12pg/ml vs 0.8pg/ml, p=0.003)]. Levels of TNFα, RANTES, IL-1β and IFNα were not different between groups. No association between cytokine concentration and age was observed in children with CF (**Figure 1H**).

### Early Life Circulating Immune Cell Proportions by Manual and Unsupervised Analysis

Whole blood samples from 16 children with CF and 3 healthy controls were available for systemic analysis (**Supplementary Table 1**). Manual gating analysis using the gating strategy outlined in **Supplementary Figure 2** was used to determine the circulating cellular immune profile of children with CF.

In children with CF, neutrophils [CD16^+^ granulocytes (17)] comprised a median of 31.9% of CD45^+^ leukocytes while eosinophils were 2.08% (**Figure 2A** and **Supplementary Figure 4A**). CD4 T cells, CD8 T cells, B cells and NK cells existed at much higher frequencies in blood relative to BAL, circulating at a median 18.24%, 6.33%, 7.5% and 3.14% of blood leukocytes, respectively. Blood monocytes could be subtyped into classical, intermediate and non-classical subsets based on CD14 and CD16 expression, with classical monocytes the most abundant (median 3.13% of leukocytes), followed by non-classical (0.42%) and intermediate (0.47%) monocytes (**Figure 2A** and **Supplementary Figure 4A**). This is distinct from BAL, where only CD16^-^ (classical) and CD16^+^ (intermediate) subsets could be identified (**Figure 1A** and **Supplementary Figure 1**). CD11c^+^ myeloid dendritic cells were also identified in whole blood, comprising a median 0.28% of leukocytes. NK-T cells were observed at 0.24% of leukocytes (**Figure 2A** and **Supplementary Figure 4A**).

As we had analysed samples from children aged 1-6, we next sought to investigate changes in cell frequency across the first five years of life in CF. Neutrophils were positively associated with age (r = 0.62, p = 0.011), whilst CD4 T cells and B cells were inversely associated with age [CD4 T (r = -0.64, p = 0.008), B cells (r = -0.71, p = 0.002)] (**Figure 2B**).

No differences in systemic immune profile were observed between CF and control children (**Figure 2C**), although we appreciate the limited numbers of control blood samples available for this study.

Clustering analysis of whole blood samples with FlowSOM using the same 13 lineage markers revealed 18 cell clusters (**Figure 2D**). Based on the expression of lineage markers, the clusters were classified into annotated cell types. This revealed clusters corresponding to three neutrophil populations, two monocyte populations (CD16^-^ and CD16^+^), two NK cell populations (CD16^-^ and CD16^+^), an undefined CD14^+^CD11b^+^ population, and one cluster corresponding to each of eosinophils, B cells, CD8 T cells and CD4 T cells. One cluster (totalling to 7.2%) was negative for all markers. The average frequencies of each cluster across all whole blood samples are shown in **Figure 2D**. Clusters corresponding to neutrophils expressed high levels of CD15 and CD16, whilst eosinophils expressed low levels of CD15 and were positive for HLA-DR and CD66b. The UMAP analysis depicted in **Figure 2E** shows that FlowSOM clusters corresponding to circulating neutrophils, eosinophils, monocytes, B cells, NK cells, CD4 T cells and CD8 T cells cluster together as expected within two-dimensional space, corresponding well to the automatically defined UMAP clusters. Cell proportions obtained from FlowSOM analysis highly correlate with those obtained using manual gating (**Supplementary Figure 4C**). UMAPs for each individual are depicted in **Supplementary Figure 4D**.

### Comparison of Inflammatory Cells Across Lung and Peripheral Blood Compartments

Granulocytes from BAL expressed higher levels of CD66b, CD11b CD15 and CD63 relative to the equivalent granulocyte population in whole blood (**Figure 2F**). Similarly, CD16^+^ monocytes from BAL expressed high levels of CD206, whilst the CD16^+^ monocytes in blood do not express this marker (**Figure 2F**).

## Discussion

In this work, we applied multi-parameter flow cytometry, inflammatory cytokine profiling, and unsupervised analytical tools to comprehensively phenotype the local and systemic immune response in children with CF and aged-matched healthy controls. We showed that children with CF have an elevated inflammatory signature in the lung characterised by activated granulocyte recruitment that is positively associated with age, and increased concentration of several key inflammatory cytokines. Systemic immune responses were similar between children with CF and healthy controls, however systemic neutrophils, CD4 T cells and B cells were associated with age across the first five years of life in children with CF. We additionally show that inflammatory cells in the BAL express higher levels of activation and migration markers relative to their systemic counterparts. While these data were derived primarily from children with CF with a small cohort of healthy controls, the methods described could be applied to any childhood respiratory disease and will assist in improving our understanding of immunity and inflammation in these conditions.

Cystic fibrosis is an exemplar condition of where understanding aberrant inflammation could lead to improvements in patient care. To date, it has been recognised that inflammation is one of the key pathophysiological processes in CF lung disease (1), however despite extensive efforts there are no effective anti-inflammatory therapies used in clinical practice. Deep immune profiling will help to identify novel therapeutic approaches which complement current care.

The most common immune cell population in the lung is the alveolar macrophage, comprising up to 90% of immune cells in the bronchoalveolar lavage fluid in children, shown by us previously as well as in the current study. Recent work has also showed distinct transcriptional profiles in macrophages obtained from foetal and adult lungs (18), highlighting that alveolar macrophages undergo significant development throughout life. Another recent finding revealed that in adults who have undergone lung transplantation, the majority of alveolar macrophages are derived from the recipient’s bone marrow, rather than from self-replenishing resident populations in the donor lung (19). Furthermore, sc-RNAseq analysis of the adult lung identified multiple alveolar macrophage subpopulations (20), a novel finding yet to be explored in children. Our unsupervised hierarchical clustering analysis revealed four alveolar macrophage subpopulations based on differences in expression of lineage markers. Future work will investigate functional differences between these subpopulations.

The value of understanding alveolar macrophages was also recently highlighted by Liao et al. who examined the sc-RNAseq profile of adults with COVID-19 infection, demonstrating that the alveolar macrophage subtype composition in BAL fluid was associated with disease severity, and that those with severe disease had higher proportions of migratory monocyte-derived macrophages which secreted IL-6 (21). This highlighted IL-6 as a therapeutic target in severe COVID-19 infection, which was supported by preliminary observational studies showing that IL-6 blockade with monoclonal antibodies improved outcomes (22). This finding has since been confirmed in larger multinational trials (23). In this setting, a better understanding of the cell composition in the lung identified potential prognostic biomarkers and therapeutic targets worthy of further evaluation.

Along with alveolar macrophages, granulocyte populations play a key role in driving inflammation in the lung. These data were derived from children with CF in whom granulocytic inflammation is a key driver of disease severity. In a seminal study, Sly et al. demonstrated the presence of free neutrophil elastase in early life BAL was the best predictor of future lung disease severity (15). In the current work, we show that children with CF have increased proportions of CD66b^+^ granulocytes.

When compared to blood granulocytes, BAL granulocytes showed higher median levels of CD66b, CD11b and CD15, all markers involved in activation and migration of granulocytes in the lung (24, 25).

We also show that monocytes make up a small proportion of immune cells in the lung and consist of two primary populations (CD16^+^ and CD16^-^). The CD16^-^ monocytes expressed the alveolar macrophage marker CD206, a feature not observed on blood monocytes. Furthermore, a subset of alveolar macrophage expresses intermediate levels of CD14. These findings suggest that in addition to the tissue-resident alveolar macrophages, there exists a population of monocyte-derived macrophages in the paediatric lung. Previous studies have shown that circulating monocytes are recruited to the lung during disease to orchestrate a pro-inflammatory immune response (26). Whether monocyte-derived macrophages are causally involved in CF inflammation will be area of future investigation. In a study of adults with COPD, peripheral blood monocytes shared an overlapping gene signature with alveolar macrophages. This signature correlated with lung function, highlighting that circulating monocytes and alveolar macrophages may both be involved in disease progression (27).

Cytokines such as TNFα (28), IL-1β (28), IL-8 (29–32), IL-6 (28, 29, 33) and MCP-1 (29, 34) have been shown to be elevated in the BAL of patients with CF when compared to controls, although these have mostly been investigated in isolation. Associations between cytokine concentration and disease severity have also been demonstrated. For example, IL-8, IL-1β and IL-1α have been associated with increased severity of structural lung disease (35, 36). We were unable to assess associations between immune profiling and disease severity as due to the challenges of measuring severity in the preschool age group, these data were not available.

A limitation of our data that must be acknowledged is the samples are derived primarily from children with CF and a small cohort of healthy control samples, which may limit the applicability to other conditions. Beyond expression of activation and migration markers, our data also do not provide insight into the function or direct clinical impact of the cell subsets described. Despite these limitations however, we have highlighted that it is possible to obtain and utilise samples collected at the time of clinically performed procedures for downstream analysis to better understand inflammation in childhood respiratory diseases.

There are several future directions that will stem from this work. Further experiments will characterise the functional impact of the cellular changes identified. In the first instance, fluorescence-activated cell sorting can be used to purify granulocyte subtypes for functional evaluation using *in vitro* stimulation experiments. In parallel to this, by employing these techniques on a larger number of samples and comparing cell type proportions to clinical outcomes (such as severity of structural lung disease measured by computed tomography), the relationship between cell subtypes and clinical disease severity can be explored. Lastly, simultaneous analysis of blood and BAL with other single cell techniques at a transcriptomic (CITE-seq) or epigenomic level (sc-ATAC-seq) would further improve our understanding of the mechanisms underlying childhood respiratory diseases.

In conclusion, the methods described in this study permit improved immune cell profiling and cytokine analysis of childhood respiratory diseases. These techniques can be applied to all childhood respiratory diseases with a potential translational impact *via* the identification of clinical biomarkers and therapeutic targets.

## Data Availability Statement

The raw data supporting the conclusions of this article will be made available by the authors, without undue reservation.

## Ethics Statement

The studies involving human participants were reviewed and approved by Royal Children’s Hospital Human Research Ethics Committee HREC #25054. Written informed consent to participate in this study was provided by the participants’ legal guardian/next of kin.

## Author Contributions

SS, SR, RS and MN contributed to the concept and design of the study. SS and SR were responsible for patient recruitment and sample collection. SS and MN performed experiments, analysed the data, and co-wrote the manuscript. MN supervised the work and interpreted the findings. MN, RS, and SR provided funding. All authors edited and approved the final manuscript.

## Conflict of Interest

The authors declare that the research was conducted in the absence of any commercial or financial relationships that could be construed as a potential conflict of interest.

## Publisher’s Note

All claims expressed in this article are solely those of the authors and do not necessarily represent those of their affiliated organizations, or those of the publisher, the editors and the reviewers. Any product that may be evaluated in this article, or claim that may be made by its manufacturer, is not guaranteed or endorsed by the publisher.
