# Supplementary material for: Mapping Pulmonary and Systemic Inflammation in Preschool Aged Children With Cystic Fibrosis
Source: Front Immunol. 2021 Oct 15;12:733217. doi: 10.3389/fimmu.2021.733217 (PMC8554310; doi:10.3389/fimmu.2021.733217)
Supplement: Supplementary file 1 [file DataSheet_1.pdf]

Supplementary Table 1. Demographics of the study cohort

Patient	Sex	Age (months)	CF or Control	No. of live CD45$^+$ cells analysed
BAL samples				
1	M	11	CF	34316
2	F	11	CF	37451
3	M	13	CF	111776
4	M	13	CF	36215
5	M	17	CF	63181
6	F	18	CF	35502
7	M	23	CF	93767
8	F	24	CF	22931
9	F	24	CF	45798
10	M	24	CF	21205
11	F	27	CF	418000
12	M	27	CF	59165
14	M	48	CF	74871
14	F	54	CF	107033
15	M	54	CF	27324
16	F	55	CF	79366
17	F	59	CF	69837
18	M	60	CF	116419
19	M	61	CF	12498
20	M	62	CF	97956
21	F	74	CF	148489
C-1	M	5	Control	21444
C-2	M	13	Control	5773
C-3	M	37	Control	156551
C-4	F	56	Control	80435
Blood samples				
1	M	11	CF	133370
2	F	11	CF	80560
3	M	13	CF	239024
4	M	17	CF	197753
5	M	23	CF	26708
6	F	24	CF	9252
7	M	24	CF	60611
8	F	27	CF	3784
9	M	27	CF	59637
10	F	54	CF	7418
11	M	54	CF	25171
12	F	55	CF	30185
13	F	59	CF	21430
14	M	60	CF	32902
15	M	62	CF	12010
16	F	74	CF	164338
C-1	M	5	Control	93392
C-2	M	13	Control	45148
C-3	F	56	Control	253831

Supplementary Table 2. Flow cytometry antibody cocktail for BAL and blood samples

**Surface Marker**	**Fluorophore**	**Clone**	**Final Dilution**
CD14	BV786	M5E2	1:50
CD11b	BUV805	ICRF44	1:100
CD45	BV711	HI30	1:100
CD56	BUV737	NCAM16.2	1:100
CD11c	PE-Cy7	B-ly6	1:100
CD63	A647	H5C6	1:100
CD4	A700	RPA-T4	1:100
CD3	BB515	UCHTI	1:100
CD15	PE-CF594	W6D3	1:200
HLADR	V500	G46-6	1:200
CD19	BV605	SJ25C1	1:200
CD8	BV650	RPA-T8	1:200
CD206	BV421	G10F5	1:200
CD66b	PE	19.2	1:200
CD16	BUV395	3G8	1:400
Live/dead	N-IR		

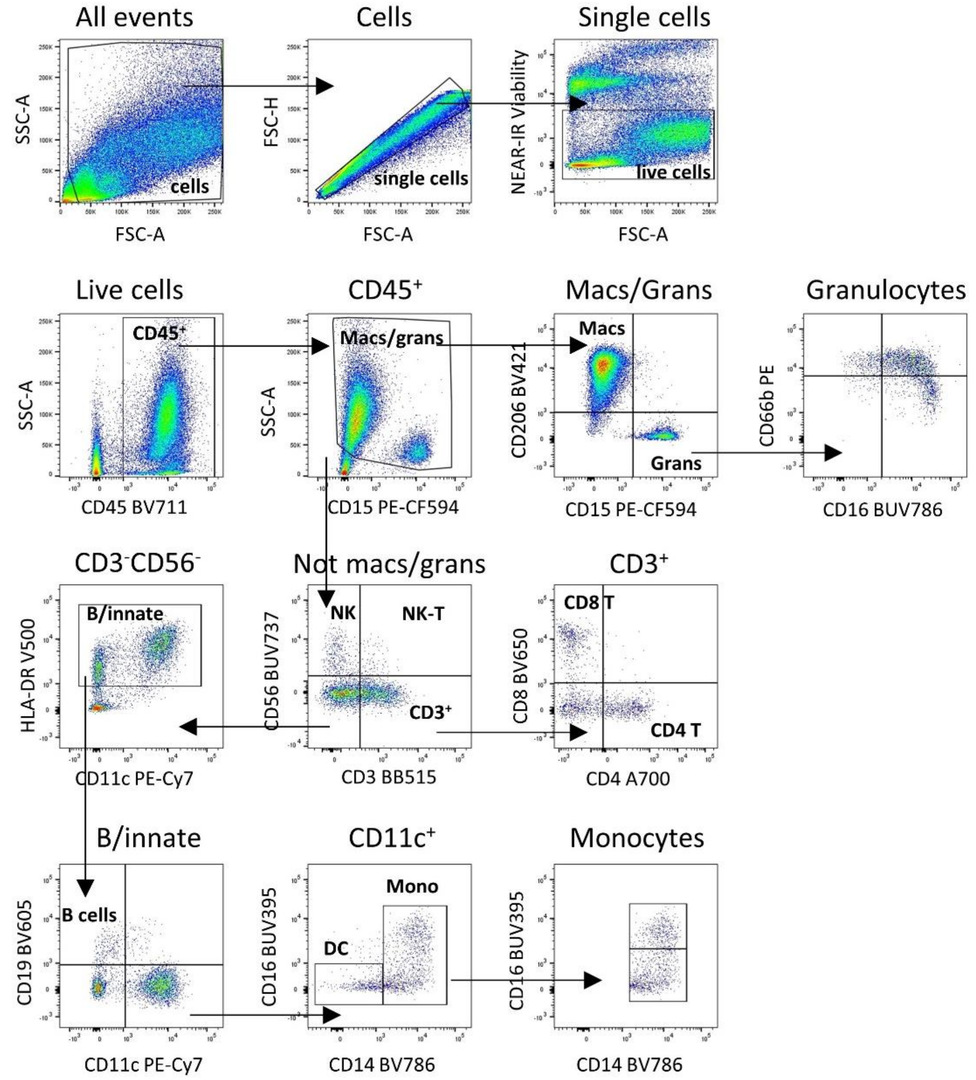

Supplementary Figure 1. Representative flow cytometry gating strategies for BAL. CD45+ leukocytes were firstly selected from the live single cell fraction. Within CD45+ cells, macrophages/granulocytes were selected based on a high SSC-A profile. Macrophages were identified based on $CD206^{+}CD15^{-}$ phenotype, whilst granulocytes were $CD206^{-}CD15^{+}$. Within the granulocyte fraction, $CD16^{+/-}$ and $CD66b^{+/-}$ granulocytes were identified. $CD45^{+}SSC\text{-}A^{low}$ cells were further subtyped into $CD56^{+}$ NK cells, $CD3^{+}$ T cells, and $CD56^{+}CD3^{+}$ NK-T cells. Within the $CD3^{+}$ T cell fraction, CD4 and CD8 T cells were identified. $HLADR^{+}CD19^{+}$ cells were identified as B cells, $HLADR^{+}CD19^{+}CD11c^{+}$ cells were identified as CD11c+ B cells, and $HLADR^{+}CD19^{-}CD11c^{+}$ cells were identified as innate cells. Within the innate cell fraction, monocytes were selected based on CD14 expression, whilst DCs were $HLADR^{+}CD11c^{+}CD14^{-}CD16^{-}$. Monocytes were also assessed for CD16 expression, revealing both $CD16^{+}$ and $CD16^{-}$ subsets.

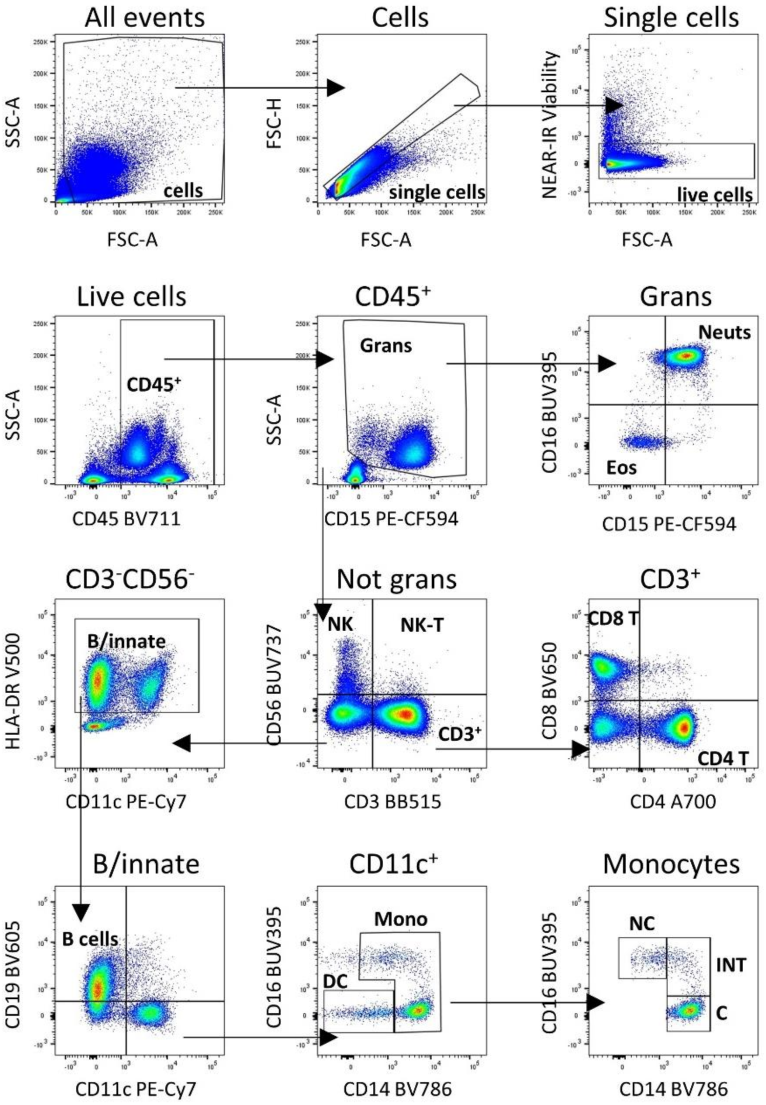

Supplementary Figure 2. Representative flow cytometry gating strategies for whole blood. CD45+ leukocytes were firstly selected from the live single cell fraction. Within CD45+ cells, granulocytes were selected based on a high SSC-A profile. Neutrophils were gated based on $CD15^{+}CD16^{+}$ expression, whilst eosinophils had low CD15 expression and were negative for CD16. $CD45^{+}SSC\text{-}A^{low}$ cells were further subtyped into $CD56^{+}$ NK cells, $CD3^{+}$ T cells, and $CD56^{+}CD3^{+}$ NK-T cells. Within the $CD3^{+}$ T cell fraction, CD4 and CD8 T cells were identified. $HLADR^{+}CD19^{+}$ cells were identified as B cells, $HLADR^{+}CD19^{+}CD11c^{+}$ cells were identified as CD11c+ B cells, and $HLADR^{+}CD19^{-}CD11c^{+}$ cells were identified as innate cells. Within the innate cell fraction, monocytes were selected based on CD14 expression, whilst DCs were $HLADR^{+}CD11c^{+}CD14^{-}CD16^{-}$. Monocytes were also assessed for CD16 expression, revealing classical ($CD14^{+}CD16^{-}$), intermediate ($CD14^{+}CD16^{+}$) and non-classical ($CD14^{low}CD16^{+}$) populations.

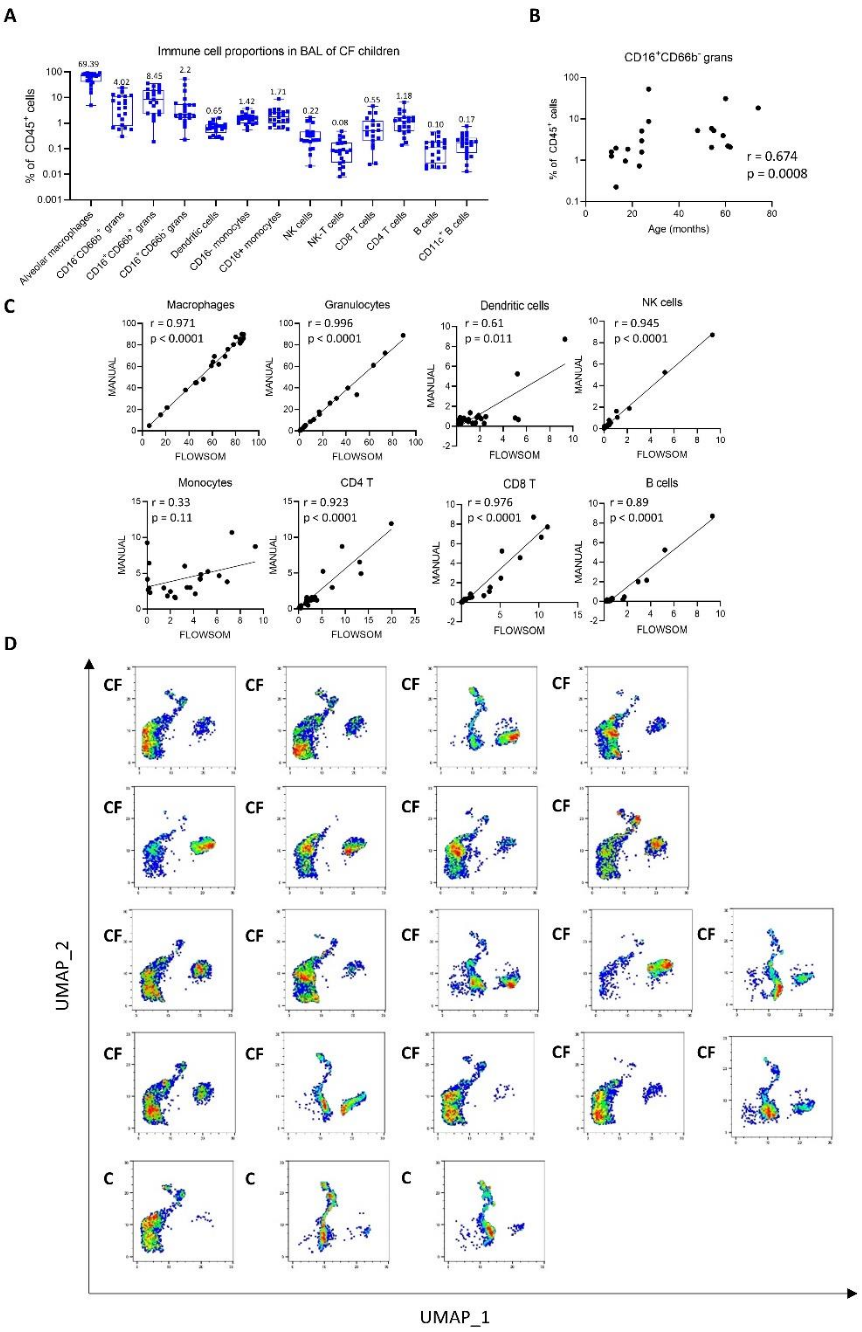

Supplementary Figure 3. Immune cell parameters in BAL. (A) Immune cell proportions in BAL of children with CF. The median for each cell population is provided. **(B)** Two-sided spearman correlation analysis of $CD16^{+}CD66b^{-}$ granulocytes and age in children with CF. **(C)** Two-sided spearman correlation analysis of cell clusters identified by FlowSOM and cell populations identified by manual gating. **(D)** Individual UMAPs for BAL samples included in unsupervised analysis.

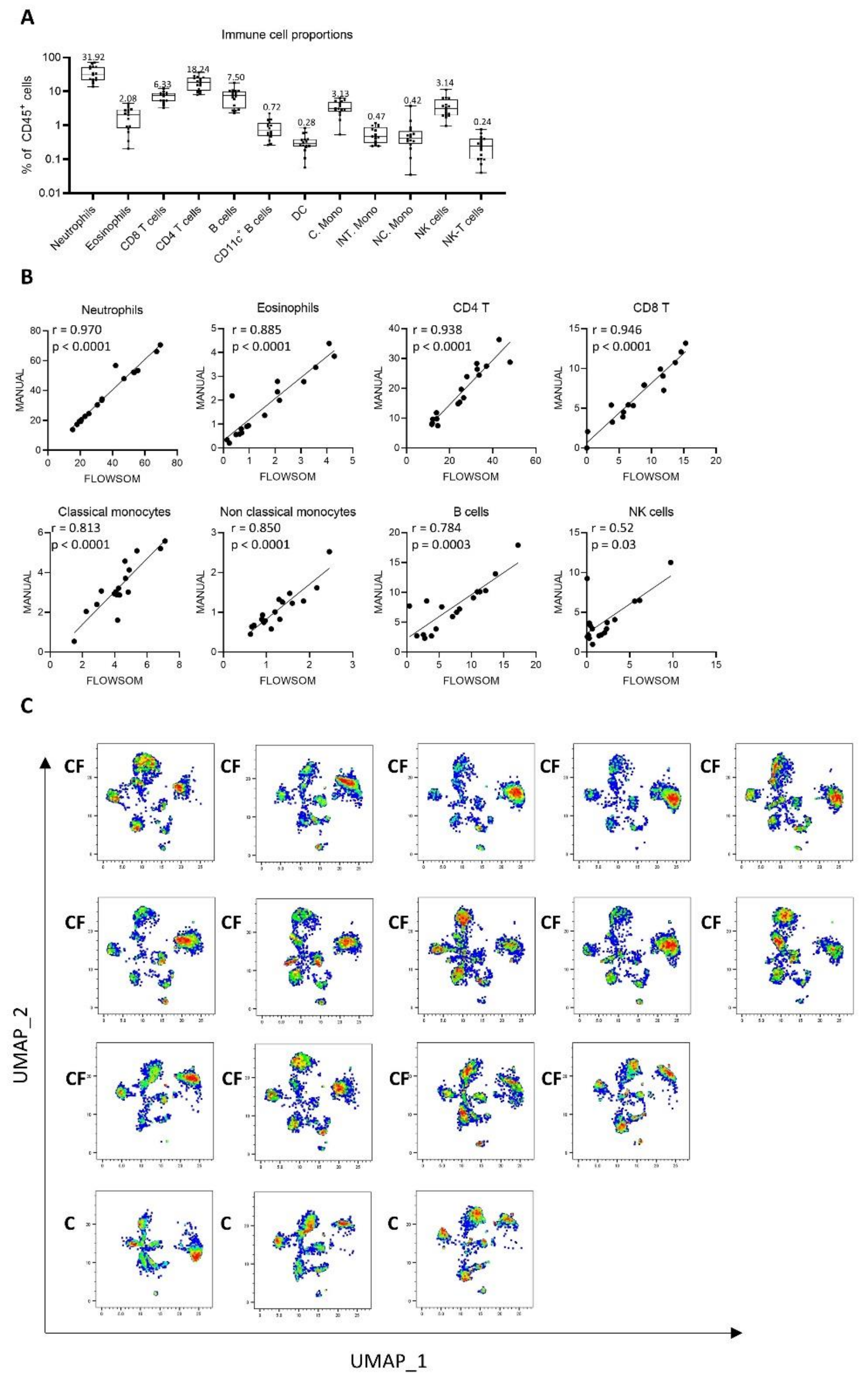

Supplementary Figure 4. Immune cell parameters in peripheral blood. (A) Immune cell proportions in blood of children with CF. The median for each cell population is provided. **(B)** Two-sided spearman correlation analysis of cell clusters identified by FlowSOM and cell populations identified by manual gating. **(C)** Individual UMAPs for BAL samples included in unsupervised analysis.
